# The hematopoietic potential of methanolic and aqueous extracts of *Portulaca oleracea *in a phenylhydrazine model of anemia

**DOI:** 10.22038/AJP.2022.20965

**Published:** 2023

**Authors:** Kobra Shirani, Bamdad Riahi-Zanjani, Seyed Navid Omidkhoda, Samira Barangi, Gholamreza Karimi

**Affiliations:** 1 *Department of Toxicology, Faculty of Medical Sciences, Tarbiat Modares University, Tehran, Iran*; 2 *Medical Toxicology Research Center, School of Medicine, Mashhad University of Medical Sciences, Mashhad, Iran*; 3 *Department of Pharmacodynamics and Toxicology, School of Pharmacy, Mashhad University of Medical Sciences, Mashhad, Iran*; 4 *Pharmaceutical Research Center, Pharmaceutical Technology Institute, Mashhad University of Medical Sciences, Mashhad, Iran*; † * Equal first author*

**Keywords:** Purslane, Phenylhydrazine, Anemia, Methanolic extract, Aqueous extract

## Abstract

**Objective:**

*Portulaca oleracea*, commonly known as Purslane, is traditionally used as a sour, diuretic, and cooling herb with hemostatic properties. The present study evaluates the antianemic effect of methanolic and aqueous extracts of *P. oleracea *in a phenylhydrazine model of anemia.

**Materials and Methods:**

Phenylhydrazine (60 mg/kg/day, i.p., two consecutive days) was used to induce anemia in rats. The aqueous and methanolic extracts of *P. oleracea* were prepared, and three methods of treatment were defined with two doses (500 and 750 mg/kg, i.p.). The hematological parameters and blood cell morphology, total and direct bilirubin, and morphology, and pathology of bone marrow were evaluated.

**Results:**

The results showed that the methanolic extract has better effects than aqueous extract in improving phenylhydrazine-induced anemia. Our results showed that administration of 500 and 750 mg/kg of *P. oleracea* methanolic extracts for 4 days could protect against the development of anemia caused by phenylhydrazine.

**Conclusion:**

In summary, the methanolic extracts of *P. oleracea* might be effective in phenylhydrazine-induced anemia.

## Introduction

Anemia is a frequent public health problem that hurts people of any age, especially females, children, and older adults of developing countries (Kassebaum, 2016 ▶). Anemia has multiple causes categorized as reduced quality or quantity of hemoglobin and/or red blood cell (RBC) count. Such low levels of hemoglobin and RBCs may decrease the ability of blood to distribute oxygen to different body organs, and thus, uncontrolled anemia may be serious or even life-threatening (Al-alimi et al., 2018 ▶). Several factors and situations are associated with anemia, such as iron and micronutrient deficiency, infectious diseases, heavy bleeding, and genetic defects. The interaction of phenylhydrazine (PHZ) with hemoglobin results in reactive oxygen species (ROS) production, which causes intensive lipid peroxidation of the erythrocytes membrane and hematotoxicity. These properties of PHZ have made it as a useful agent in experimental model studies of hemolytic anemia  (Ashour, 2014 ▶). 

There are different types of treatments for hemolytic anemia, including administration of iron, vitamin B12 or B9, treatment with immune-suppressors or corticosteroids, erythropoietin injection, blood transfusion, and bone marrow stem cell transplant (Zanella and Barcellini, 2014 ▶). Innovating and discovering therapeutic agents from safe sources has been receiving considerable attention of pharmacologists due to the crucial role that could be played by herbal medicine in prophylaxis and/or therapy of diseases, as well as improving the health status and performance of normal subjects (Ansari-Mohseni et al., 2022 ▶; Gholamnezhad et al., 2019 ▶). The low cost, availability, accessibility, and effectiveness are some reasons of the widespread use of medicinal plants (Shirani et al., 2020). One of the most valuable herbal remedies, which is traditionally used for alleviating several diseases, is *Portulaca oleracea. *It is an annual plant weed belonging to the Portulacaceae family, with extensive distribution throughout the world, including Mediterranean countries, Africa and Asia (Chan et al., 2000 ▶; Uddin et al., 2014 ▶). It was mentioned with the name of *Andrachne*, *Khorfeh*, *Porcilaca*, *Purslane*, and *Portulaca* in different traditional medicine and herbal remedies books. In ethno-medicine, *P. oleracea *is considered a sour, diuretic, antidiabetic, and calmative agent. In excessive menstrual flow, stomachache, gout, colds, headache and inflammation of male genitalia, the juice of the plant is beneficial (Foutami et al., 2020 ▶). The Traditional Persian Medicine recommended *P. oleracea *as a medication for severe inflammations, kidneys and bladder pains, erysipelas, fevers, insomnia, blepharitis, mouth ulcers, eye pain, gastritis, intestinal ulcers, cough, tonsillitis, and asphyxia (Iranshahy et al., 2017 ▶; Radhakrishnan et al., 2001 ▶; Sultana and Rahman, 2013 ▶). In folk medicine,* P. oleracea *is considered to have blood cooling and hemostatic properties to treat bleeding bacillary dysentery, hemorrhage and leucorrhea, hematochezia, hemorrhagic vomiting, hemoptysis, bleeding haemorrhoids, blood purification, eruption of blood, abnormal uterine bleeding and metrorrhagia (Sultana and Rahman, 2013 ▶). 

*P. oleracea* has high nutritional value and contains high levels of antioxidants such as omega-3 fatty acids and phenolic compounds, mineral, coumarins, cardiac and anthraquinone glycosides, and alkaloids (Petropoulos et al., 2016 ▶). Recent studies have reported some pharmacological activities from *P. oleracea* such as muscle relaxant activity, reduction in locomotor activity, anticonvulsant, gastro-protective action, analgesic, anti-inflammatory, antioxidant, hepatoprotective, analgesic, and wound healing activities, and its effect on hyperthyroidism, and hypochloresterolemic (Iranshahy et al., 2017 ▶; Kaveh et al., 2017 ▶; Khanam et al., 2019 ▶; Khodadadi et al., 2018 ▶; Rahimi et al., 2019 ▶). Here, we attempted to survey the therapeutic effect of methanolic and aqueous extracts of *P. oleracea *on the PHZ-induced anemia. 

## Materials and Methods

**Plant material**

*P. oleracea* (herbarium number: 12-1615-240) was collected in March 2019 from private fields of Mashhad University of Medical Sciences, Mashhad, Iran. The aerial parts of plant were washed and dried in the open air under the shade for 48 hr and stored at room temperature. The dried *P. oleracea* was ground by using a mixer grinder (Moligrano; Becken). 

**Extraction and isolation**

One hundred grams of the powdered plant was extracted by 1000 ml of methanol and water using the maceration method for 48 hr. During this time, the mixture was stirred intermittently. The solutions were then filtered through Whatmann filter paper. To prepare the dry extract, the obtained solutions were dried by a rotary evaporator and their solvent was separated. These extracts were dried of water and solvent by freeze dryer. The yield of aqueous and methanolic extract were 19% and 13%, respectively. Then, they were stored at -20°C until used (Jafarian et al., 2014 ▶). 

**Experimental design**

Ninety adult female Wistar rats weighing 180–200 g were provided by Animal House, School of Pharmacy, Mashhad University of Medical Sciences, Mashhad, Iran and kept at 20±4°C, humidity of 10%, and a 12-hr light/dark cycle with free access to food and water. This study was done in 2020 at the School of Pharmacy, Mashhad University of Medical Sciences, Mashhad, Iran. All the experiments were performed according to Mashhad University of Medical Sciences, Ethical Committee Acts (IR.MUMS.REC.1396.55).

**Figure 1:**
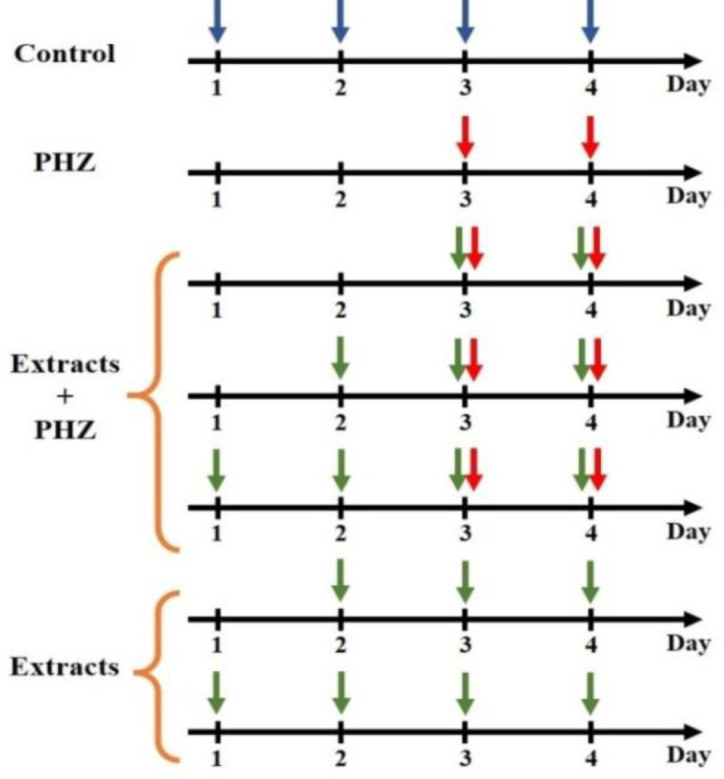
The summary of different groups and diverse compounds in the first, second, third, and fourth days of treatment. The Blue arrow shows normal saline. The red arrow shows phenylhydrazine. The green arrow shows the aqueous or methanolic extract of *P. oleracea*

Animals were randomly divided into eighteen groups, with five rat per group according to Table 1. In control group, rats received normal saline (intraperitoneally; i.p.) for 4 consecutive days. Anemia was induced by i.p. injection of PHZ (60 mg/kg) for 2 days (Sang Hun et al., 2012 ▶). In treatment groups, animals received i.p. injection of different doses (500 or 750 mg/kg) of aqueous or methanolic extracts of *P. oleracea *with or without of PHZ. The summary of different groups, which received diverse compounds during the treatment days was shown in Figure 1.

**The effect of aqueous and methanolic extracts of** ***P. oleracea*** **on hematological parameters**

Blood samples were collected from rats by cardiac puncture and collected in sterile tubes that were coated with K2-EDTA (Ethylenediaminetetraacetic acid) as an anticoagulant. The hematology test was performed using the SysmexKX-21N automated hematology analyzer (Sysmex Corporation, Kobe, Japan). 

**The effect of aqueous and methanolic extracts of** ***P. oleracea*** **on bilirubin levels in serum**

At the end of the treatment, the blood was collected through the cardiac puncture in gel and clot activator tubes. The blood samples were centrifuged for 15 min at 2000 g to prepare serum and maintained at -70°C until testing. The serum obtained from the blood samples was used to measure bilirubin (total and direct) using the Cobas Integra 400 Clinical Chemistry Analyzer (Roche, USA)(Arthur et al., 2012 ▶).

**The effect of aqueous and methanolic extracts of** ***P. oleracea*** **on blood cell morphology**

For each sample, a blood smear was prepared manually by the wedge-spread film technique and stained using the May-Grünwald & Giemsa stains. Manual blood smear review and differential leukocyte counting were carried out in all samples according to the recommendations for the grading of peripheral blood cell morphological features. Of course, in this regard, a semi-quantitative determination manner for the smear review was selected. Changes in blood cells' morphological characteristics were expressed in three levels; 

Level I: the morphological state is normal, Level II: a mild abnormality is seen. This level refers to changes in size, shape, and colors of the RBCs (such as anisocytosis, hypochromia, marocytes, microcytes and polychomasia, and so on), if these changes are observed in less than 10 % of total RBC populations, 

Level III: the damage is evident. This level refers to changes in size, shape, and colors of the RBCs if these changes are observed in more than 10-20 % of total RBC populations (Palmer et al., 2015 ▶). 

**The effect of aqueous and methanolic extracts of** ***P. oleracea*** **on bone marrow**

After animal sacrificing, femurs of each rat were collected and smears of bone marrow were prepared and stained with hematoxylin and eosin (H&E). The results were qualitatively expressed in four levels; 

Level I: bone marrow cellularity is below 60% and normal (>40% lipid tissue)

 Level II: bone marrow cellularity is 60-75% (25-40% lipid tissue) 

Level III: bone marrow cellularity is 75-90% (10-25% lipid tissue) 

Level IV: bone marrow  is highly activated and cellularity is 90-95% (5-10% lipid tissue) (Bolliger, 2004).

**Statistical analysis**

Statistical calculations of data were performed by the GraphPad Prism 7.0 software (GraphPad Prism Software Inc., San Diego, CA, USA). All parametric data are presented as means±SD. One-way analysis of variance (ANOVA) and Tukey-Kramer post hoc test were used to compare different groups at the end of the treatment. Statistical differences were considered to be significant at p<0.05.

## Results

**The effect of aqueous and methanolic extract of** ***P. oleracea*** **on hematological parameters**

Significant reductions in RBC, hemoglobin, and hematocrit levels were induced in the rats treated with PHZ compared to the normal saline group (Table 2 and 3). Different doses (500 and 750 mg/kg) of the aqueous extract could not improve hematological changes induced by PHZ (Table 2). In the case of methanolic extract, only the dose of 750 mg/kg two days before PHZ administration increased the levels of RBC, hemoglobin, hematocrit and MCH index compared to the PHZ group (Table 3).

**The effect of aqueous and methanolic extract of** ***P. oleracea*** **on bilirubin levels**

PHZ treatment significantly increased the levels of total and direct bilirubin compared to normal saline control (Table 4 and 5). The results of rat pretreatment with aqueous extract of *P. oleracea *demonstrated no significant changes in total or direct bilirubin level compared to the PHZ group (Table 4). On the other hand, in groups received the methanolic extract, the treatment of methanolic extract at 500 and 750 mg/kg for 4 days, induced a significant decrease in total and direct bilirubin level in comparison to PHZ group (Table 5).

**Table 1:** The experimental design for the evaluation of protective effect of methanolic and aqueous extracts of *P. oleracea* on PHZ-induced anemia in rats (n=5/group)

	**Groups**	**First day**	**Second day**	**Third day**	**Fourth day**
1	Control	Normal saline	Normal saline	Normal saline	Normal saline
2	PHZ	-------	-------	PHZ (60 mg/kg )	PHZ (60 mg/kg )
3	ME 500 mg/kg + PHZ	-------	-------	ME (500 mg/kg)+PHZ (60 mg/kg )	ME (500 mg/kg)+PHZ (60 mg/kg )
4		-------	ME (500 mg/kg)	ME (500 mg/kg)+PHZ (60 mg/kg )	ME (500 mg/kg)+PHZ (60 mg/kg )
5		ME (500 mg/kg)	ME (500 mg/kg)	ME (500 mg/kg)+PHZ (60 mg/kg )	ME (500 mg/kg)+PHZ (60 mg/kg )
6	ME 750 mg/kg + PHZ	-------	-------	ME (750 mg/kg)+PHZ (60 mg/kg )	ME (750 mg/kg)+PHZ (60 mg/kg )
7		-------	ME (750 mg/kg)	ME (750 mg/kg)+PHZ (60 mg/kg )	ME (750 mg/kg)+PHZ (60 mg/kg )
8		ME (750 mg/kg)	ME (750 mg/kg)	ME (750 mg/kg)+PHZ (60 mg/kg )	ME (750 mg/kg)+PHZ (60 mg/kg )
9	ME 750 mg/kg	-------	ME (750 mg/kg)	ME (750 mg/kg)	ME (750 mg/kg)
10		ME (750 mg/kg)	ME (750 mg/kg)	ME (750 mg/kg)	ME (750 mg/kg)
11	AE 500 mg/kg + PHZ	-------	-------	AE (500 mg/kg)+PHZ (60 mg/kg )	AE (500 mg/kg)+PHZ (60 mg/kg )
12		-------	AE (500 mg/kg)	AE (500 mg/kg)+PHZ (60 mg/kg )	AE (500 mg/kg)+PHZ (60 mg/kg )
13		AE (500 mg/kg)	AE (500 mg/kg)	AE (500 mg/kg)+PHZ (60 mg/kg )	AE (500 mg/kg)+PHZ (60 mg/kg )
14	AE 750 mg/kg + PHZ	-------	-------	AE (750 mg/kg)+PHZ (60 mg/kg )	AE (750 mg/kg)+PHZ (60 mg/kg )
15		-------	AE (750 mg/kg)	AE (750 mg/kg)+PHZ (60 mg/kg )	AE (750 mg/kg)+PHZ (60 mg/kg )
16		AE (750 mg/kg)	AE (750 mg/kg)	AE (750 mg/kg)+PHZ (60 mg/kg )	AE (750 mg/kg)+PHZ (60 mg/kg )
17	AE 750 mg/kg	-------	AE (750 mg/kg)	AE (750 mg/kg)	AE (750 mg/kg)
18		AE (750 mg/kg)	AE (750 mg/kg)	AE (750 mg/kg)	AE (750 mg/kg)

PHZ:Phenylhydrazine, ME:Methanolic extract, AE:Aqueous extract

**The effect of aqueous and methanolic extracts of** ***P. oleracea*** **on blood cell morphology**

**Figure 2:**
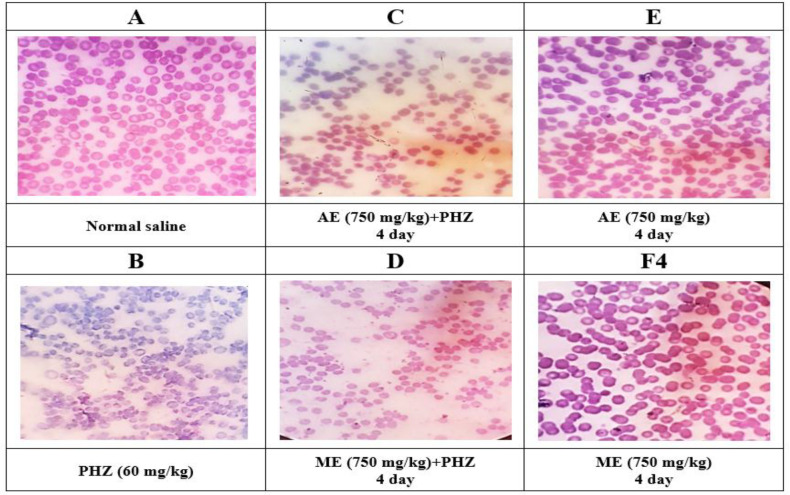
Representative pictures of blood cell morphology from different treatment groups stained with hematoxylin and eosin (H&E) examined under a microscope (magnification: ×100). The changes in red blood cells such as hypochromia, anisocytosis and poikilocytosis in the blood morphology of rats were examined. The results were reported qualitatively at three levels for PHZ group (level 3), groups receiving aqueous and methanolic extracts with PHZ (level 2) and normal saline and extracts control groups (level 1). PHZ=Phenylhydrazine; ME=Methanolic Extract; AE=Aqueous Extract

The investigation of blood smear in different groups showed that PHZ administration induced morphological changes in RBC including hypochromic, anisocytosis and poikilocytosis (Figure 2B). Both aqueous and methanolic extracts of *P. oleracea* (750 mg/kg) were able to reduce these changes when administered to rats two days before induction of anemia (Figure 2C and D). Normal saline and extracts alone caused no morphological changes in treated animals (Figure 2A, E and F). 

**The effect of aqueous and methanolic extracts of** ***P. oleracea*** **on bone marrow**

**Figure 3:**
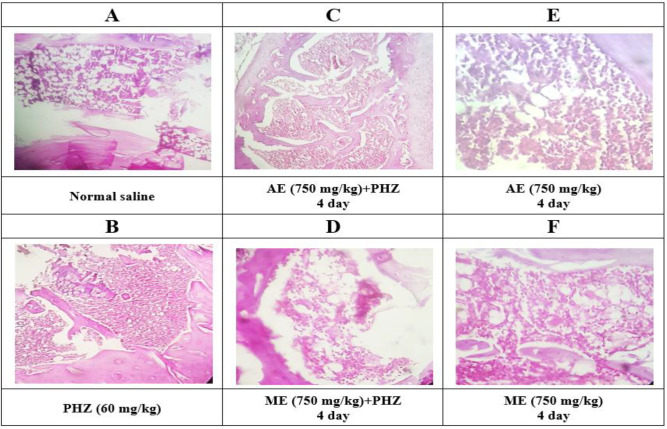
The histopathological changes of bone marrow morphology in different groups stained with hematoxylin and eosin (H&E) examined under a microscope (magnification: ×40). Aqueous and methanolic extracts of *P. oleracea* were given to animals two days before anemia. In both groups, the volume of hypercellularity reduced compared to the PHZ group and had a protective effect. The results are expressed qualitatively at four levels. At level four, the bone marrow is highly activated and cellularity is between 90-95 % and tissue lipids level is between 5-10 %. At level three, cellularity level is between 90-75%, at the second level 75-60% and at the level one and normal below 60%. Level 4 for PHZ group, level 1 for normal saline as well as methanolic and aqueous extracts groups, level 3 for extract groups with PHZ are reported. PHZ=Phenylhydrazine; ME=Methanolic Extract; AE=Aqueous Extract

The investigation of bone marrow histopathological changes in different groups showed that tissue cellularity in normal saline and extracts groups (750 mg/kg) was below 60% (level I, Figure 3A, E and F). As shown in Figure 3B, PHZ administration caused anemia, activated bone marrow and increased cell division, which in turn increased cellularity (90-95%) and decreased lipid volume (Level IV). Pretreatment with aqueous and methanolic extracts (750 mg/kg) of *P. oleracea *two days prior to PHZ injection reduced these damages to level III (Figure 3C and D).

**Table 2:** The effect of aqueous extract of *P. oleracea* on hematological parameters in different groups

**Groups** **Factors**	**Normal saline**	**PHZ**	**2 days**		**3 days**		**4 days**		**3 days** **(750 mg/kg)**	**4 days** **(750 mg/kg)**
			**500 mg/kg+PHZ**	**750 mg/kg+PHZ**	**500 mg/kg+PHZ**	**750 mg/kg+PHZ**	**500 mg/kg+PHZ**	**750 mg/kg+PHZ**		
**RBC ** **(×10** ^6^ **/µl)**	6.3±0.2	2.9±0.4^###^	3.3±0.6	3.4±0.5	2.8±1.0	3.1±0.7	2.7±1.1	2.7±0.6	5.0±0.7	4.4±0.7
**HGB ** **(g/dl)**	12.9±0.5	7.4±1.4^###^	8.0±0.2	7.3±0.6	7.5±0.8	7.0±0.9	6.4±0.4	6.1±0.5	8.3±0.4	8.2±0.8
**HCT ** **(%)**	37.8±1.7	17.7±2.4^###^	21.3±1.6^*^	18.4±0.4	18.5±1.1	17.3±0.6	17.4±1.3	15.9±1.4	28.8±1.9	28.6±2.1
**MCV ** **(fl)**	60.2±1.6	57.3±3.1	56.9±1.2	57.7±1.4	56.1±1.1	54.5±1.2	55.5±1.7	53.4±0.3^*^	57.5±1.5	58.9±1.3
**MCH ** **(pg)**	21.0±0.8	25.3±0.5^###^	23.9±0.9	24.2±0.6	24.1±0.6	23.9±0.4	23.9±0.7	24.1±0.5	21.1±0.7	21.4±0.8

Data were analyzed by one-way ANOVA following Tukey-Kramer post test for multiple comparisons. Data are expressed as mean±SD, (n=5). ^###^p<0.001 vs. normal saline group, ^*^p<0.05 vs. PHZ group. PHZ:Phenylhydrazine, RBC:Red blood cell, HGB:Hemoglobin, HCT:Hematocrit, MCV:Mean corpuscular volume, MCH:Mean corpuscular hemoglobin

**Table 3:** The effect of methanolic extract of *P. oleracea* on hematological parameters in different groups

**Groups** **Factors**	**Normal saline**	**PHZ**	**2 days**		**3 days**		**4 days**		**3 days** **(750 mg/kg)**	**4 days** **(750 mg/kg)**
			**500 mg/kg+PHZ**	**750 mg/kg+PHZ**	**500 mg/kg+PHZ**	**750 mg/kg+PHZ**	**500 mg/kg+PHZ**	**750 mg/kg+PHZ**		
**RBC ** **(×10** ^6^ **/µl)**	7.8±0.8	3.3±0.04^###^	3.4±0.2	3.6±0.2	3.6±0.2	3.9±0.3	4.1±0.4	5.2±0.2^**^	7.8±0.5	6.8±0.7
**HGB ** **(g/dl)**	13.6±1.1	6.9±0.7^###^	7.1±0.5	7.3±0.3	7.6±0.2	7.8±0.3	10.1±0.8	11.2±0.9^*^	12.3±0.9	13.2±1.4
**HCT ** **(%)**	39.6±1.0	16.4±1.6^###^	16.5±0.5	16.8±0.9	17.2±0.7	18.7±0.8	22.3±1.2	26.1±1.5^*^	36.1±2.5	38.8±3.6
**MCV ** **(fl)**	50.4±0.4	48.9±2.1	49.4±2.3	48.8±1.2	48.7±1.9	49.4±0.9	55.6±2.3	55.5±2.1	50.2±1.4	56.9±1.5
**MCH ** **(pg)**	17.3±0.3	20.8±0.6^###^	20.7±0.6	20.5±0.6	20.3±0.8	19.7±0.6	22.9±1.1	22.1±1.2^*^	17.5±0.3	19.4±0.7

Data were analyzed by one-way ANOVA following Tukey-Kramer post test for multiple comparisons. Data are expressed as mean±SD, (n=5). ^###^p<0.001 vs. normal saline group, ^*^p<0.05 and ^**^p<0.01 vs. PHZ group. PHZ:Phenylhydrazine, RBC:Red blood cell, HGB:Hemoglobin, HCT:Hematocrit, MCV:Mean corpuscular volume, MCH:Mean corpuscular hemoglobin

**Table 4:** The effect of aqueous extract of *P. oleracea* on total and direct bilirubin levels in different groups

**Groups** **Factors**	**Normal saline**	**PHZ**	**2 days**		**3 days**		**4 days**		**3 days** **(750 mg/kg)**	**4 days** **(750 mg/kg)**
			**500 mg/kg+PHZ**	**750 mg/kg+PHZ**	**500 mg/kg+PHZ**	**750 mg/kg+PHZ**	**500 mg/kg+PHZ**	**750 mg/kg+PHZ**		
**Bili. T ** **(m** **g/dl)**	0.09±0.01	0.36±0.02^###^	0.30±0.03	0.29±0.05	0.28±0.06	0.27±0.02	0.27±0.05	0.26±0.04	0.09±0.02	0.07±0.0
**Bili. D ** **(m** **g/dl)**	0.05±0.01	0.27±0.01^###^	0.25±0.01	0.24±0.01	0.23±0.02	0.23±0.03	0.22±0.03	0.22±0.03	0.06±0.01	0.05±0.0

Data were analyzed by one-way ANOVA following Tukey-Kramer post test for multiple comparisons. Data are expressed as mean±SD, (n=5). ^###^p<0.001 vs. normal saline group. PHZ:Phenylhydrazine, Bili T:Bilirubin total, Bili D:Bilirubin direct

**Table 5:** The effect of methanolic extract of *P. oleracea* on total and direct bilirubin levels in different groups

**Groups** **Factors**	**Normal saline**	**PHZ**	**2 days**		**3 days**		**4 days**		**3 days** **(750 mg/kg)**	**4 days** **(750 mg/kg)**
			**500 mg/kg+PHZ**	**750 mg/kg+PHZ**	**500 mg/kg+PHZ**	**750 mg/kg+PHZ**	**500 mg/kg+PHZ**	**750 mg/kg+PHZ**		
**Bili. T ** **(m** **g/dl)**	0.09±0.01	0.53±0.03^###^	0.52±0.02	0.50±0.02	0.49±0.02	0.48±0.02	0.41±0.08^**^	0.38±0.07^***^	0.08±0.01	0.06±0.01
**Bili. D ** **(m** **g/dl)**	0.06±0.01	0.31±0.04^###^	0.30±0.03	0.28±0.03	0.26±0.03	0.25±0.02	0.20±0.04^**^	0.18±0.04^***^	0.05±0.01	0.04±0.01

Data were analyzed by one-way ANOVA following Tukey-kramer post test for multiple comparisons. Data are expressed as mean±SD, (n=5). ^###^p<0.001 vs. normal saline group, ^**^p<0.01 and ^***^p<0.001 vs. PHZ group. PHZ:Phenylhydrazine, Bili T:Bilirubin total, Bili D:Bilirubin direct

## Discussion

PHZ is a chemical widely used to develop hemolytic anemia in animals. In this study, to ensure the anemia-producing dose of PHZ, a pilot experiment was performed at the dose of 60 mg/kg which showed more severe anemia comprising erythrocytopenia, reduced hemoglobin, and reduced mean corpuscular volume (MCV) index accompanied by higher indices of mean corpuscular hemoglobin (MCH) after two days of i.p. injection. As a result of anemia and destruction of RBC, bilirubin released from the blood cells and the serum levels of total bilirubin increased in PHZ treated groups (Phillips and Henderson, 2018 ▶). The hematotoxic action of PHZ is mainly attributed to its ability to cause free radicals production, lipid peroxidation, oxidative degradation of spectrin in cell membrane, and lysis of erythrocytes, which lead to hemolysis and anemia (Pandey et al., 2018 ▶). Reactive oxygen species (ROS) production by PHZ was related to the binding of oxidized and denatured hemoglobin to the RBC membrane. As a result, PHZ-induced hemolysis appears to be caused by oxidative changes in red blood cell proteins rather than membrane lipids (Berger, 2007 ▶). Thus, prevention of oxidative stress could be a strategy against PHZ-induced hemolytic anemia. Plant-derived antioxidants are well known for their ability to shield the cellular systems against damage raised as a result of oxidative stress (Shukla and Singh, 2015 ▶).

The morphology of RBC in the peripheral blood smear reflects its function because deformability is an essential criterion for circulating RBC (Huisjes et al., 2018 ▶). Sharma et al. demonstrated that PHZ caused accentuated degenerative changes such as acanthocytes, schistocytes, anisocytosis and poikilocytosis in circulating RBC in acute hemolytic anemia (Sharma et al., 1991 ▶). In our study, peripheral blood smear images also showed changes including hypochromia, anisocytosis and poikilocytosis in PHZ-treated erythrocytes, while aqueous and alcoholic extracts of *P. oleracea* were able to protect erythrocyte deformities, which indicates the decreased deformation of erythrocytes or recovery of morphological status.

PHZ toxicity leads to hemolytic anemia, which is known to be accompanied by changes in hematological parameters such as RBC count, hemoglobin, hematocrit, MCV and MCH (Shukla et al., 2012 ▶). Memisoglu et al. showed hemoglobin and hematocrit decrease as well as total and direct bilirubin increase in rats received PHZ (Memisoglu et al., 2017 ▶). Moreover, PHZ treatment in rats resulted in a remarkable decrease in RBC count, hemoglobin, hematocrit and MCV, while platelets level, MCH and MCHC enhanced in PHZ group (Ousaaid et al., 2022 ▶). The elevation of MCH seems to be induced by a free plasma haemoglobin increase (Berger, 2007 ▶). Our results in line with previous studies results revealed the reduction in RBC count, hemoglobin, hematocrit and MCV parameters and increase in MCH in rats received PHZ, which were reversed by pretreatment of *P. oleracea* methanolic extract.

In this study, we evaluated the anti-anemia potential of aqueous and methanolic extracts of *P. oleracea *on PHZ-induced anemia in rats and findings showed that methanolic extract performed better than the aqueous extract. The antioxidant activities of *P. oleracea* extracts have been previously demonstrated. A study by YouGuo et al. (2009) ▶ revealed that polysaccharides compounds of *P. oleracea* could significantly scavenge superoxide anion, 2,2-diphenyl-1-picrylhydrazyl (DPPH), nitric oxide, and hydroxyl radicals in a dose-dependent manner (YouGuo et al., 2009 ▶). According to a previous study, the protective activity of betacyanins of *P. oleracea *against D-galactose-induced neurotoxicity was due to the increase in the activities of antioxidant enzymes with a reduction in lipid peroxidation (Wang and Yang, 2010 ▶). Antioxidant activities of three phenolic alkaloids isolated from *P. oleracea* were based on the scavenging activity against DPPH radicals and inhibitory effect on hydrogen peroxide‐induced lipid peroxidation in rat brain (Erkan, 2012 ▶). 

The antioxidant potential of *P. oleracea* could be explained by its rich content of omega-3 fatty acids, gallotannins, kaempferol, quercetin, apigenin, α-tocopherols, glutathione, ascorbic acid, vitamin A, and B-complex vitamins. These compounds protect cells as powerful antioxidants by preventing or repairing free radicals species-induced damages (Simopoulos et al., 1992 ▶; Zhou et al., 2015 ▶).

In the current study, the pretreatment with methanolic extract showed better effects than aqueous extract which is supported by other studies showing that the total flavonoid and phenols content and antioxidant activity of nonpolar extracts of *P. oleracea* such as dichloromethane, and methanol, are higher than polar extracts (Zhu et al., 2009 ▶).

In summary, the results of our study demonstrated that the preventive method in the use of *P. oleracea* extract had a better effect in improving PHZ-induced hemolytic anemia and also its methanolic extract showed better anti-anemia effects than its aqueous extract. However, more studies are needed in this area. 

## Conflicts of interest

The authors have declared that there is no conflict of interest.
